# The FAAH Inhibitor URB597 Modulates Lipid Mediators in the Brain of Rats with Spontaneous Hypertension

**DOI:** 10.3390/biom10071022

**Published:** 2020-07-10

**Authors:** Michał Biernacki, Marta Baranowska-Kuczko, Gabriella N. Niklińska, Elżbieta Skrzydlewska

**Affiliations:** 1 Department of Analytical Chemistry, Medical University of Bialystok, Mickiewicza 2D, 15-222 Bialystok, Poland; michal.biernacki@umb.edu.pl; 2 Department of Experimental Physiology and Pathophysiology, Medical University of Bialystok, Mickiewicza 2A, 15-222 Bialystok, Poland; marta.baranowska@umb.edu.pl; 3 Department of Large Animal Diseases and Clinic, Institute of Veterinary Research Centre and Center for Biomedical Research, Warsaw University of Life Science, 100 Nowoursynowska St., 02-797 Warsaw, Poland; Gabriella.N.Niklinska@vetmed.uni-giessen.de

**Keywords:** spontaneous hypertension, lipid peroxidation products, endocannabinoid system, URB597, rat brain

## Abstract

Hypertension is accompanied by oxidative stress, which can be modified by the functioning of the endocannabinoid system playing a prominent modulatory role in the brain. The present study tested whether chronic administration of the fatty acid amide hydrolase (FAAH) inhibitor [3-(3-carbamoylphenyl) phenyl]N-cyclohexylcarbamate (URB597) to rats with primary hypertension (SHR) can modify redox balance and consequently brain phospholipid metabolism. Experiments were conducted using SHRs and normotensive control Wistar–Kyoto rats treated by intraperitoneal injection with URB597 for 14 days. The biochemical parameters were assayed in the rats’ brains. Inhibition of FAAH activity by URB597 resulted in an increase in anandamide and GPR55 receptor levels, as well as a decrease in CB_2_ receptor expression. However, there was a simultaneous increase in Nrf2 expression, as well as Cu, Zn-SOD, GSH-Px, glutathione reductase activity, and vitamin E levels in brain tissue of SHR rats. Consequently, URB597 caused a decrease in levels of phospholipid fatty acids and MDA, and an increase in free fatty acids. Given the importance of maintaining redox balance for brain function, the results of this study point to endocannabinoids as a potential therapeutic target for preventing brain metabolic disorders in hypertension.

## 1. Introduction

Hypertension is an important risk factor for cerebrovascular diseases, including stroke. Hypertension also plays an important role in the development of vascular cognitive impairment and vascular dementia [1]. Hypertension is typically associated with an increase in the generation of reactive oxygen species (ROS) in the central nervous system (CNS) that control blood pressure [2]. Physiological levels of ROS are important for maintaining proper homeostatic functioning of brain cells, whereas ROS overload can exceed the antioxidant abilities and lead to oxidative stress [3]. The major source of ROS in brain cells is NADPH oxidase [4]. NADPH oxidase activity has been shown to be enhanced by activation of AT1 receptors in animals with hypertension [4]. Hypertension also promotes a reduction in activity of antioxidant enzymes such as Cu-Zn-SOD, CAT, GSH-Px, and GSH level [4,5]. The transcription factor Nrf2 plays an important role in maintaining the proper level of antioxidant proteins. Nrf2 is responsible for the biosynthesis of cytoprotective antioxidant proteins, but expression of Nrf2 is reduced in the brains of hypertensive rats [6]. In addition, Nrf2 expression strongly correlates with mitochondrial function by promoting the mitochondrial biogenesis pathway [7]. The brain tissue is also characterized by a high content of polyunsaturated fatty acids (PUFAs), which increases susceptibility to oxidative modifications [8]. Consequently, an increased production of lipid peroxidation products has been observed in the paraventricular nucleus of the hypothalamus and in the cerebellar vermis of hypertensive rats [4,5]. Subsequent research has demonstrated that antioxidant therapy promotes the reduction of blood pressure in the hypothalamus of spontaneously hypertensive rats (SHRs), and that these effects are mediated by reduced oxidative stress and inflammation [5].

Recent work has highlighted the critical role of the endocannabinoid system in regulating levels of ROS and TNF-α and thus the modulation of oxidative stress and inflammation [9]. In addition, changes in endocannabinoid levels and the expression of endocannabinoid receptors in various tissues have been demonstrated in several pathophysiological conditions, including hypertension [10,11]. However, endocannabinoid levels, particularly anandamide (AEA), have been shown to depend on activity of the fatty acid amide hydrolase (FAAH). FAAH is a membrane-associated enzyme that is responsible for the degradation of endocannabinoids [12]. Therefore, the endocannabinoid system is an attractive therapeutic target for the treatment of hypertension and other cardiovascular diseases [9,13]. Given that AEA levels have been shown to regulate blood pressure [13], FAAH inhibitors are thought to be potential antihypertensive agents. Previous studies in SHRs demonstrated that acute administration of the FAAH inhibitors URB597 [14] and AM3506 [15] results in a normalization of blood pressure and a decrease in cardiac contractility. The chronic administration of URB597 to DOCA-salt hypertensive rats has been shown to decrease cardiac and renal hypertrophy in an age-dependent manner [16]. Moreover, it was shown that chronic administration of URB597 disturbs redox balance through a decrease in ROS levels and changes in antioxidants abilities. These effects, in turn, result in lipid and protein oxidative modifications in the heart, liver, and kidney of hypertensive rats [10,11,17].

Changes in endocannabinoid levels can also affect the metabolism and functioning of brain cells [18]. Other studies show that activation of the cannabinoid type 2 (CB_2_) receptor is associated with reduced ROS generation in a variety of tissue types, including the brain [19]. On the other hand, activation of the cannabinoid type 1 (CB_1_) receptor promotes ROS production and macrophage inflammatory responses, including p38MAPK activation [20]. In contrast, the neuroprotective role of the CB_1_ receptor has been documented in a mouse model of Parkinson’s disease [21]. This neuroprotection by nonselective CB_1_ agonists (WIN55,212-2 and HU210) was accompanied by increased survival of nigrostriatal dopaminergic neurons in the striatum, suppression of NOX and ROS production, and reduced expression of pro-inflammatory cytokines in activated microglia cells [21]. Other studies demonstrate that AEA protects hippocampal neurons from oxidative injuries by decreasing intracellular ROS levels and lowering the expression of NOX2 in a CB_1_ receptor-mediated manner [22].

Animal models (e.g., SHRs) can be used to evaluate the pathophysiology and pharmacotherapy of brain damage caused by hypertension, and these models correspond with primary hypertension in humans. Therefore, the purpose of this study was to evaluate the effects of chronic administration of the FAAH inhibitor URB597 on the redox system in the brain, and in subsequent modifications of phospholipid metabolism in rats with spontaneous hypertension.

## 2. Material and Methods

### 2.1. Materials

Drugs and reagents were obtained from the following sources: 3-(3-carbamoylphenyl)phenyl N-cyclohexylcarbamate (URB597), 8-iso Prostaglandin F_2α_-d_4_ (8-isoPGF2α–d_4_), 8-iso Prostaglandin F_2α_ (8-isoPGF2α) anandamide-d_8_ (AEA-d_8_), and 2-arachidonylglycerol-d_8_ (2-AG-d_8_) from Cayman Chemical Company (Ann Arbor, MI, USA); dimethyl sulfoxide (DMSO), superoxide dismutase (SOD), catalase (CAT), L-ascrobic acid, retinol, L-glutathione reduced (GSH), and Tween 80 were acquired from Sigma-Aldrich (Steinheim, Germany); pentobarbital sodium was purchased from Biowet (Puławy, Poland), chloro-2,4-dinitro benzene (CDNB), butylated hydroxytoluene (BHT), and 5,5′-dithiobis (2-dinitrobenzoic acid) (DTNB) were acquired from Sigma-Aldrich (Steinheim, Germany). URB597 was dissolved in an URB597 solvent: a mixture of DMSO, Tween 80, and saline (0.9% NaCl) [1:2:7; *v*/*v*/*v*].

### 2.2. Animals

The experiment was performed using 8–10-week-old male (270–350 g) rats with primary hypertension (i.e., SHRs) and normotensive control Wistar–Kyoto rats (WKY). Rats were maintained in a 12:12 h light–dark cycle with free access to standard pelleted rat chow and tap water. This study was conducted in accordance with the guidelines of the European Directive (2010/63/EU). The animal care and protocols in this study were reviewed and approved by the local Animal Ethics Committee in Białystok (Poland) [resolution No. 4/2012 of 25.01.2012]. According to the European Directive, every effort has been made to reduce the number of animals used and to minimize animal suffering during the experiment.

### 2.3. Experimental Protocol

The experimental protocol was conducted according to the guidelines of the European Directive (2010/63/EU) and was approved by the local Animal Ethics Committee in Białystok (Poland) [resolution No. 4/2012 of 25.01.2012].

The rats were divided into 4 different groups of six rats each:

- Group 1 [WKY]: WKY rats were treated intraperitoneally (*i.p.*) with URB597 solvent [1 mL—mixture of DMSO, Tween 80 and saline (0.9% NaCl) [1:2:7; *v*:*v*:*v*]] every 12 h, during the last 14 days;
- Group 2 [WKY + URB597]: WKY rats were treated *i.p.* with URB597 [1 mg/kg b.w. in 1 mL of URB597 solvent] every 12 h, during the last 14 days;
- Group 3 [SHR]: SHRs were treated *i.p.* with URB597 solvent [1 mL] every 12 h, during the last 14 days;
- Group 4 [SHR + URB597]: SHRs were treated *i.p.* with URB597 [1 mg/kg b.w. in 1 mL of URB597 solvent] every 12 h, during the last 14 days.

In conscious rats, systolic blood pressure (SBP) was measured using the tail-cuff method before and after URB597 (or solvent) treatment. Rats with SBP values ≥ 150 mmHg were considered hypertensive. The two-week URB597 administration did not alter SBP in SHR (187 ± 15 and 191 ± 49 mmHg) or WKY (117 ± 18 and 101 ± 10 mmHg) rats before the first and the final dose, respectively. The solvent for URB597 did not alter SBP either in the SHR (184 ± 34 and 205 ± 43 mmHg) or WKY (114 ± 18 and 110 ± 13 mmHg) rats before the first and final injections, respectively [11].

### 2.4. Tissue Preparation

All surgical procedures were performed after *i.p.* injection of pentobarbital (70 mg/kg b.w.). At the end of the experiment, rats were sacrificed and the brain was removed and weighed, followed by washing with isotonic saline (40 °C). Then, the brains were divided sagittally into the left and right hemispheres:

1. The right hemisphere was pulverized in liquid nitrogen to examine levels of endocannabinoids, fatty acids and their metabolites, GSH, and FAAH activity;
2. The left hemisphere after washing with isotonic saline (4 °C) was homogenized under standardized conditions to obtain 10% homogenates in 0.9% NaCl solution, which were centrifuged at 20,000× *g* for 15 min at 4 °C. Supernatants were used for the determination of various biochemical parameters (vitamins A and E, Cu, Zn-SOD, GSH-Px, GSSG-R, Nrf2, Keap1, Bach1, p62, HO-1, CB_1_, CB_2_, GPR55).

### 2.5. Methods

#### 2.5.1. Antioxidant Enzymes Activity

Superoxide dismutase (Cu, Zn–SOD—EC.1.15.1.1) activity was assayed by the oxidation of epinephrine. Oxidation of epinephrine is measured by the production of adrenochrome, which exhibits an absorption maximum at 480 nm [23]. One unit of SOD was defined as the amount of the enzyme that inhibits epinephrine oxidation to adrenochrome by 50%. Enzyme-specific activity was expressed in U per milligram of protein.

Glutathione peroxidase (GSH-Px—EC.1.11.1.6) activity was assayed by the conversion of NADPH to NADP^+^. This conversion is accompanied by a decrease in absorbance at 340 nm [24]. One unit of GSH-Px activity was defined as the amount of enzyme catalyzing the oxidation of 1 mmol NADPH per minute. Enzyme-specific activity was expressed in U per milligram of protein.

Glutathione reductase (GSSG-R—EC.1.6.4.2) activity was assayed by assessing the oxidation of NADPH. Oxidation of NADPH is associated with a decrease in absorbance at 340 nm [25]. One unit of GSSG-R was defined as the amount of enzyme that catalyzes the oxidation of 1 mmol of NADPH per minute. Enzyme-specific activity was expressed in U per milligram of protein.

#### 2.5.2. Western Blot Analysis

Western blot analysis of cellular proteins (Nrf2, Keap1, Bach1, p62, HO-1, CB_1_, CB_2_, GPR55) was performed according to Eissa and Seada [26]. Primary antibodies raised against Keap1 (host: goat), Nrf2 and β-actin (host: mouse), and GPR55 (host: rabbit) were purchased from Sigma-Aldrich and used at a concentration of 1:1000. Primary antibodies against Bach1, p62, CB_1_, and CB_2_ (host: rabbit) were purchased from Santa Cruz Biotechnology and used at a concentration of 1:1000. Whole brain homogenates or membrane fractions containing 30 µg proteins were mixed with sample loading buffer (Laemmle buffer containing 5% 2-mercaptoethanol), heated at 95 °C for 10 min, and separated by 10% Tris-glycine SDS-PAGE. The same procedure was used to prepare the negative control (containing pure PBS buffer) and the positive control (commercially purchased complete cell lysate—Santa Cruz Biotechnology, Santa Cruz, CA, USA). As internal loading controls, β-actin and Na+/K+ ATPase (for brain homogenates and membrane fractions, respectively) were used. Following separation, proteins were electrophoretically transferred onto nitrocellulose membranes. The blotted membranes were blocked with 5% skim milk in TBS-T buffer (5% Tween 20) for 1 h and cut into fragments corresponding to the selected molar masses of the proteins. Visualized protein bands were quantitated using the Versa Doc System and Quantity One software. The results are expressed as a percentage of the expression determined in the control groups.

#### 2.5.3. Non-Enzymatic Antioxidants Level

The level of reduced glutathione (GSH) was quantified using capillary electrophoresis (CE) (PA 800 plus, Beckman Coulter, Brea, CA, USA) [27]. The separation was performed on a capillary with 50 cm total length (40 cm effective length) and 50 µm i.d. and was operated at 27 kV with UV detection at 200 ± 10 nm. The GSH concentration was determined from the calibration curve over a range: 0.1–100 µmol/L (*r*^2^ = 0.9989). The levels of GSH are expressed as µmol per gram tissue.

High-performance liquid chromatography (HPLC-1260 Infinity, Agilent Technologies, Santa Clara, CA, USA) was used to determine the concentration of vitamins A and E [28]. Vitamins A and E were extracted from homogenates using hexane. The hexane phase was removed, dried, and diluted in ethanol, and 50 µL of the mixture was injected into the column. Analyses of vitamins were performed on an RP-18 column with UV detection at 294 nm. The flow rate was 1 mL/min of methanol and water (95:5). The concentration of each vitamin was determined using a calibration curve ranging from 0.1–10 nmol/L for vitamin A (*r*^2^ = 0.9997), and from 1–200 nmol/L for vitamin E (*r*^2^ = 0.9998). The levels of vitamins are expressed as nM per gram tiisue.

#### 2.5.4. Phospholipid and Free Fatty Acids and Their Metabolism

Levels of phospholipid and free fatty acids were measured using gas chromatography (GC) (Clarus 500, Perkin-Elmer, Waltham, MA, USA) [29]. Fatty acids were extracted using Folch extraction with a chloroform/methanol mixture (2:1, *v/v*) in the presence of 0.01% butylated hydroxytoluene. Free fatty acids and total phospholipids were separated using thin-layer chromatography (TLC) with the mobile phase: heptane–diisopropyl ether–acetic acid (60:40:3, *v*/*v*/*v*). All lipid fractions were converted to fatty acid methyl esters (FAMEs) using transmethylation with boron trifluoride in methanol reagent under nitrogen atmosphere. Derivatives of fatty acids were analyzed using GC with a flame ionization detector (FID) (Perkin Elmer, Clarus 500). Separation of FAMEs was carried out on a capillary column coated with Varian CP-Sil88 stationary phase (50 m × 0.25 mm, ID 0.2 µm, Varian). Nonadecanoic acid (19:0) and 1,2-dinonadecanoyl-*sn*-glycero-3-phosphocholine (19:0 PC) were added as internal standards. Fatty acid concentrations were determined using the following calibration curve ranges: 20–10,000 µg/mL for the phospholipids arachidonic acid (AA) and docosahexaenoic acid (DHA); 1–500 µg/mL for phospholipids linoleic acid (LA); 1–500 µg/mL for free AA and DHA; and 0.1–100 µg/mL for free AA. Correlation coefficients curves for phospholipids AA, DHA, and LA, and free AA, DHA, and LA were *r*^2^ = 0.9997, *r*^2^ = 0.9998, *r*^2^ = 0.9999, *r*^2^ = 0.9997, *r*^2^ = 0.9998, and *r*^2^ = 0.9999, respectively. The levels of phospholipid and free fatty acids are expressed as µmol per gram tissue.

Lipid peroxidation was determined by measuring the levels of malondialdehyde (MDA) by GC-MS/MS GC-7000 quadrupole MS/MS, Agilent Technologies, Santa Clara, CA, USA) [30] and as F_2_-isoprostanes (8-isoPGF_2α_) by LC-MS/MS (LCMS 8060, Shimadzu, Kioto, Japan) [31].

MDA was derivatized by the addition of O-(2,3,4,5,6-pentafluoro-benzyl) (PFB) hydroxylamine hydrochloride. Derivatized aldehyde was analyzed using a 7890A GC—7000 quadrupole MS/MS (Agilent Technologies, Palo Alto, CA, USA) equipped with a HP-5ms capillary column (0.25-mm internal diameter, 0.25-µm film thickness, 30-m length). The column temperature was initially set at 50 °C for 1 min, increased by 10 °C/min until 200 °C, then raised by 3 °C/min until 220 °C, raised by 20 °C/min until 310 °C, and maintained at 310 °C for 5 min. The injector temperature was maintained at 250 °C, the transfer line was kept at 280 °C, and the source temperature was set to 230 °C. MDA was detected by the selected ion-monitoring (SIM) mode. Quantitation was achieved using an internal standard (benzaldehyde-D6). The following ions were used: *m/z* 204.0 and 178.0 for MDA-PFB and *m/z* 307.0 for IS (benzaldehyde-D6) derivatives. The level of MDA is expressed as µM per gram tissue.

8-isoPGF_2α_ was extracted using solid phase extraction (SPE) using ultra-performing liquid chromatography tandem mass spectrometry (LCMS 8060, Shimadzu, Kioto, Japan). A gradient separation was performed starting with a solvent that consisted of 60% H_2_O (pH 3.85) and 40% ACN running for 4 min. In 1 min, a gradient was run to 100% acetonitrile which ran till 8 min. From 8 to 9 min, a gradient was used to return to the original eluent composition and the system was equilibrated till 15 min. 8-isoPGF_2α_ was analyzed in negative-ion mode using MRM mode. 8-isoPGF_2α_-d_4_ was used as internal standards for quantification. Transitions of the precursor to the product ion was as follows: *m/z* 353.2→193.1 for 8-isoPGF_2__α_ and 357.2→197.1 for 8-isoPGF_2__α_-d_4_. The level of 8-isoPGF_2α_ is expressed in nanograms per gram tissue.

Levels of the endocannabinoids AEA and 2-arachidonylglycerol (2-AG) were estimated using ultra-performing liquid chromatography tandem mass spectrometry ((LCMS 8060, Shimadzu, Kioto, Japan) [32]. The initial chromatographic conditions are 70% of ACN in water containing 0.1% (*v/v*) of formic acid as the ionizing agent. After isocratic development for 1 min; a gradient is applied up to 80% ACN from min 1–5, followed by a second gradient up to 88% ACN from min 5 to 15; then, 100% ACN is reached after 0.5 min. These conditions are kept constant until the end of the chromatographic step that finishes at min 25. Endocannabinoids were extracted using solid phase extraction (SPE) analyzed in the positive-ion mode using the MRM mode. AEA-d_8_ and 2-AG-d_8,_ were used as internal standards for quantification. Transitions of the precursor to the product ion was as follows: *m/z* 348.3→62.1 for AEA, *m/z* 379.3→287.2 for 2-AG, 356.3→63.1 for AEA-d_8_, and *m/z* 387.0→295.0 *m/z* for 2-AG-d_8_. Endocannabinoid levels are expressed in nmoles or pmoles per gram of tissue.

FAAH (EC 3.5.1.99) activity was measured in the brain homogenate prepared in 20 mM Tris, containing 10% glycerol, 150 mM NaCl, and 1% Triton X-100, pH 7.8 at 4 °C. After centrifugation [1000× *g*], 20 µL of the supernatant was added to 175 µL of reaction buffer (125 mM Tris, pH 9.0, and 1 mM EDTA) and 17 µM FAAH substrate decanoyl mnitroaniline (m-NA). FAAH activity was defined as the amount of *m*-nitroaniline (m-NA) released from decanoyl *m*-nitroaniline and evaluated spectrophotometrically at 410 nm [33]. Enzyme-specific activity was expressed in nmoles of m-NA/min/mg protein.

### 2.6. Statistical Analysis

The data are expressed as mean ± standard deviation (SD). Statistical comparisons were performed by two-way analysis of variance (ANOVA) followed by a post hoc Tukey test. The results were considered statistically significant if the *p* values were 0.05 or less.

## 3. Results

**Figure 1:**
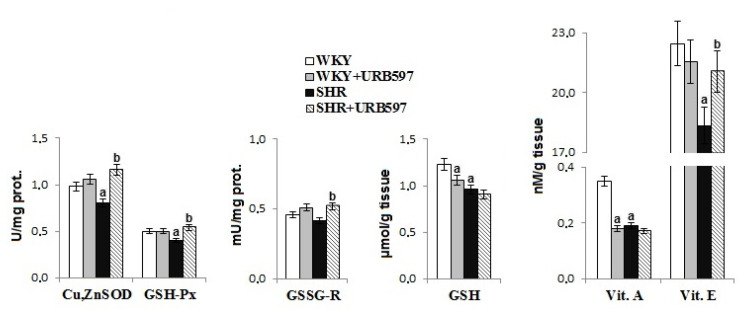
The activities/levels of antioxidant parameters in the brain of rats with spontaneous hypertension and hypertensive rats after URB597 administration. Data points represent the mean ± SD, *n* = 6; (a, significantly different from the Wistar–Kyoto (WKY) group, *p* < 0.05; b, significantly different from the of spontaneously hypertensive rats (SHR) group, *p* < 0.05).

Primary hypertension led to reduced antioxidant activity in the rat brain tissue (Figure 1). There was a reduction in the activity of antioxidant enzymes (i.e., Cu, Zn-SOD, GSH-Px) and the levels of all non-enzymatic antioxidants (GSH, vitamins E and A) in the brain of hypertensive rats. However, administration of the FAAH inhibitor, URB597, partially prevented the observed alterations, causing an increase in activity of Cu, Zn-SOD, GSH-Px, and GSSG-R, as well as an increase in vitamin E levels. Administration of the FAAH inhibitor also decreased the levels of GSH and vitamin A in normotensive control WKY rats.

**Figure 2:**
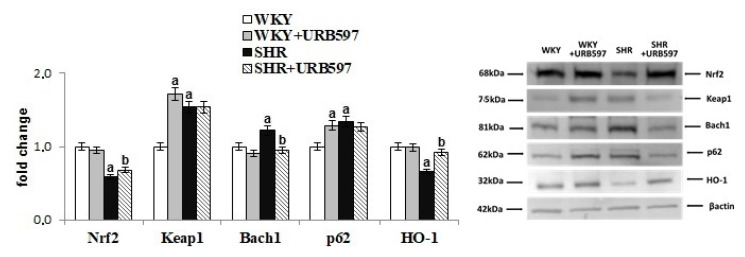
The levels of Nrf2 and its activator (p62) and inhibitors (Keap1, Bach1) as well as HO-1 in the brain of spontaneously hypertensive rats and hypertensive rats after URB597 administration. The expression of the examined proteins is shown compared to the control groups. Data points represent the mean ± SD, *n* = 6; (a, significantly different from the WKY group, *p* < 0.05; b, significantly different from the SHR group, *p* < 0.05).

We found that hypertension altered antioxidant defenses at the transcriptional level estimated by changes in the transcription factor Nrf2 and its activator and inhibitors’ expression (Figure 2). Indeed, primary hypertension was associated with increased expression of Nrf2 inhibitors, such as Keap1 and Bach1, which in turn, was correlated with low expression of Nrf2 and HO-1. However, the expression of the Nrf2 activator, p62 was increased in hypertensive rats. The opposite effects were observed after the chronic administration of URB597 to rats with primary hypertension resulted in an increase in levels of Nrf2 and HO-1, and a decrease in levels of Bach1. URB597 given to normotensive rats caused an increase in the expression of Keap1 and p62.

**Figure 3:**
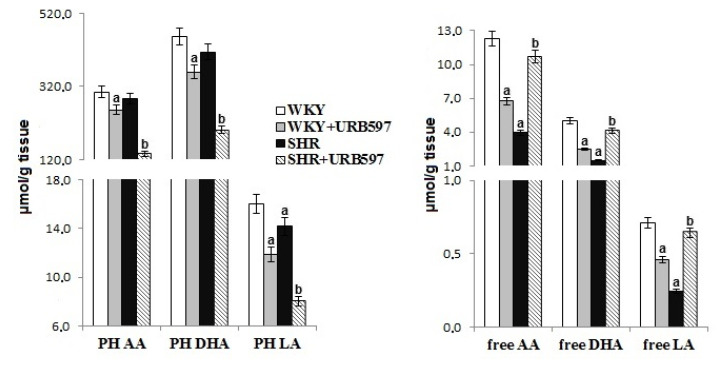
The levels of phospholipid and free fatty acids in the brain of spontaneously hypertensive rats and hypertensive rats after URB597 administration. Data points represent the mean ± SD, *n* = 6; (a, significantly different from the WKY group, *p* < 0.05; b, significantly different from the SHR group, *p* < 0.05).

**Figure 4:**
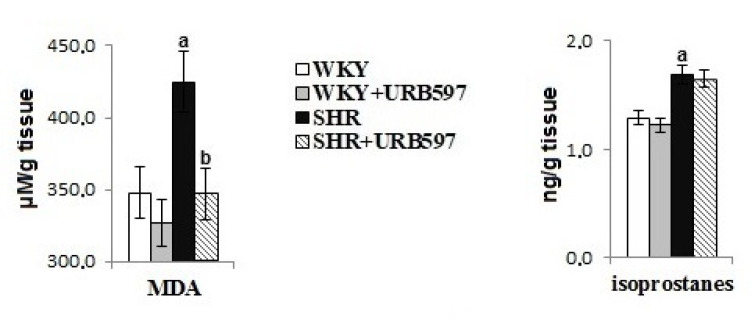
The levels of lipid peroxidation products (MDA and 8-isoPGF_2_) in the brain of spontaneously hypertensive rats and hypertensive rats after URB597 administration. Data points represent the mean *± SD*, *n* = 6; (a, significantly different from the WKY group, *p* < 0.05; b, significantly different from the SHR group, *p* < 0.05.

Primary hypertension modified brain phospholipid metabolism, evidenced by observed levels of phospholipid and free fatty acids and lipid peroxidation products (Figure 3 and Figure 4). Primary hypertension led to a decrease in the level of free PUFAs (AA, DHA, LA) and phospholipid LA. Oxidative stress favored oxidative phospholipid damages evidenced by enhanced levels of lipid peroxidation products (MDA and 8-isoprostanes). Administration of URB597 to hypertensive rats led to a decrease in phospholipid fatty acid levels, as well as an increase in free fatty acid levels. In contrast, in normotensive rats, we observed the opposite pattern of changes in phospholipid and free fatty acids following injection of URB597. In SHRs, only the level of MDA was shown to decrease after administration of URB597.

**Figure 5:**
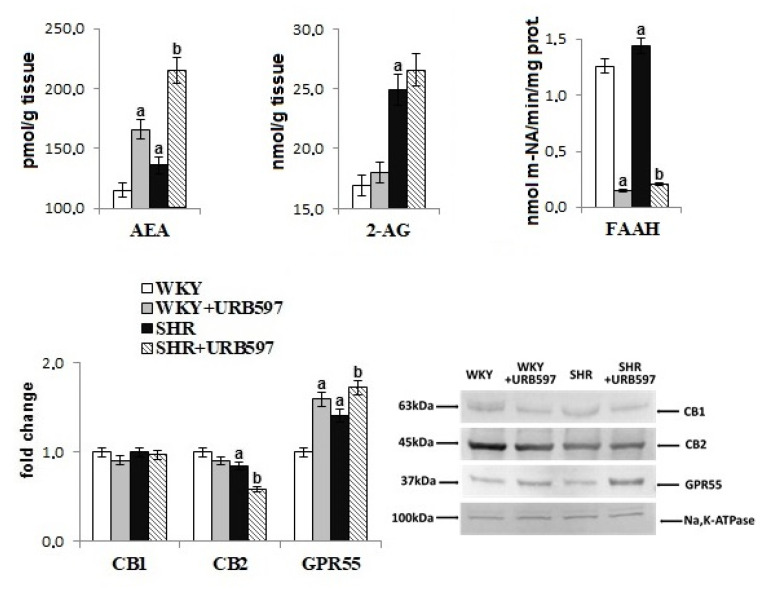
The level of endocannabinoids (AEA, 2-AG) and activity of enzyme-degrading endocannabinoids (FAAH) as well as expression of receptors (CB_1_, CB_2_, GPR55) in the brain of spontaneously hypertensive rats and hypertensive rats after URB597 administration. The expression of the examined proteins is shown compared to the control groups. Data points represent the mean ± SD, *n* = 6; (a, significantly different from the WKY group, *p* < 0.05; b, significantly different from the SHR group, *p* < 0.05).

Changes in brain phospholipid metabolism induced by hypertension were also associated with disturbances in the endocannabinoid system (Figure 5). Despite the increased activity of the endocannabinoid-degrading enzyme FAAH, the levels of AEA and 2-AG were significantly increased in the brain of hypertensive rats. GPR55 receptor expression was enhanced, whereas expression of the CB_2_ receptor was decreased in hypertensive rats. The administration of URB597 caused further increase in AEA levels and the expression of GPR55, and a decrease in FAAH activity in both groups of rats (normotensive and hypertensive). Moreover, expression of CB_2_ decreased in the brain of hypertensive rats after URB597 administration.

## 4. Discussion

Hypertension is associated with redox imbalance that has previously been observed in the blood of patients with hypertension, as well as in the blood, heart, lungs, kidneys, and brain of rats with spontaneous hypertension [10,11,34,35,36]. At 20% of total O_2_ consumption in the body, the high metabolic rate of the brain and the content of free iron ions promote the excessive production of ROS. Coupled with low levels of antioxidants, the high ROS levels lead to oxidative stress and, consequently, to oxidative modifications of cellular components [37]. In addition, there is a high content of phospholipids in the brain that contain polyunsaturated fatty acids, which are extremely susceptible to ROS. These phospholipids additionally make the brain vulnerable to oxidative modifications and, consequently, to metabolic disorders [8]. To-date, other studies have shown the presence of oxidative lipid and protein modification products in the brain of SHRs [35,36]. Thus, hypertension, which is accompanied by redox imbalance in the brain, may not only be a major risk factor for cerebrovascular problems such as stroke and cerebral hemorrhage, but may also promote the development of several neurological disorders (e.g., Alzheimer’s disease, Parkinson’s disease, Huntington’s disease, amyotrophic lateral disease, and cerebellar ataxia) [38]. Therefore, studies that assess the impact of potential hypertension therapies must also consider the effect of these therapies on redox balance and metabolic changes in brain tissue.

Redox balance, particularly in brain tissue, has been shown to depend largely upon antioxidant capacity. Results of the present study are consistent with prior published studies. In particular, we found reduced levels of non-enzymatic antioxidants (reduced levels of glutathione, and vitamins A and E), as well as reduced activity of antioxidant enzymes such as superoxide dismutase (Cu, Zn-SOD) and glutathione peroxidase (GSH-Px) in the brain of SHRs. These antioxidants are involved in the elimination of ROS. Glutathione peroxidase and lipophilic vitamins have been shown to be [4,5] effective for actively protecting membrane phospholipids against peroxidation. Reduction in the level of non-enzymatic antioxidants is thought to arise from the oxidation of these compounds by ROS, given that ROS production is typically elevated in hypertension [4]. As a result, non-enzymatic antioxidants that work, in part, to support the protection of phospholipids in the brain [39] may be unable to effectively fulfill this role. In addition, reduced levels of GSH—which also acts as a glutathione peroxidase co-substrate—are not conducive to the reduction of peroxides (e.g., lipid peroxides) [39]. As a result, there may be an increase in lipid peroxidation products, such as MDA and 8-isoPGF_2α_, as observed in this study. However, the decrease in the activity of the studied antioxidant enzymes may be the result of reduced expression and efficiency of the Nrf2 transcription factor. Nrf2 is responsible for the transcription of genes of cytoprotective proteins, including antioxidants [40]. A reduction in Nrf2 may result from elevated levels of the Nrf2 inhibitors cytosolic Keap1 and nuclear Bach1. Elevated levels of Keap1 promote the ubiquitination of Nrf2 and thus direct this transcription factor to proteasomal degradation [41]. Consequently, the reduced number of Nrf2 molecules is involved in the transcription of antioxidants. In addition, the Nrf2 nuclear inhibitor Bach 1 is also upregulated, which further prevents the transcription of Nrf2 genes [42]. As a result, there is a decrease in protein levels and activity of antioxidant enzymes. In addition, literature data indicate that the effectiveness of the Nrf2 pathway can also modulate mitochondrial ROS production in primary cultures of glioneuronal and brain lobes [43]. Regardless of the transfection activities of Nrf2, the reduction in the effectiveness of antioxidative enzymes may also result from oxidative modifications of these proteins molecules under oxidative conditions, as previously indicated in the literature [6,35].

Brain tissue is strongly enriched in n-6 PUFA, arachidonic acid, n-3 PUFA, and docosahexaenoic acid, which are necessary for optimal brain function [44]. The mechanism of action of PUFAs is still poorly understood and is further complicated by the diverse repertoire of bioactive lipid mediators that are generated during physiological and pathophysiological metabolism in brain tissue [45]. The results of this study also confirm that hypertension promotes the modification of oxidative PUFAs that occur through both oxidative fragmentation with MDA production, as well as oxidative cyclization with the production of 8-iso-prostaglandin-F2α. Changes in the structure of membrane phospholipids can directly destroy the integrity of biomembranes and lead to significant changes in the biophysical functions of brain tissue [46]. However, generated peroxidation products can be dangerous to other components of the brain tissue. For example, MDA has been found to be neurotoxic and may impair brain function [47]. Elevated levels of this neurotoxic lipid peroxidation product have been shown in the blood serum and erythrocytes of hypertensive patients [34,48]. The most commonly accepted biomarker for lipid peroxidation and indirectly oxidative stress is 8-iso-prostaglandin F2α (8-iso-PGF2α). 8-iso-PGF2α is the product of the oxidative cyclization of arachidonic acid, and elevated levels of -iso-PGF2α were observed in this study. Elevated 8-iso-PGF2α levels have also been reported in earlier studies [49,50]. Oxidative stress is hypothesized to be both a cause and a consequence of high blood pressure [2].

Regardless of ROS-dependent oxidative metabolism, PUFAs, especially in pathological conditions such as hypertension, undergo extensive enzymatic metabolism [51,52]. As a result of the action of lipases, fatty acids are metabolized to form various products, including endocannabinoids. This metabolism involves arachidonic acid, in particular, which is a fatty acid that is found in the largest amounts in the brain tissue. Importantly, endocannabinoids are the basic elements of the cannabinoid system that regulate various biological functions, including functions within the brain [53]. Other studies show that physiological stimuli lead to the rapid and transient release of endocannabinoids, which activate CB_1_ neuronal receptors, modulate ion channels, and inhibit neurotransmission [54]. Pathological conditions, in contrast, lead to much slower and sustained increases in endocannabinoid tone that result in changes in gene expression. In pathological conditions, endocannabinoids also induce molecular mechanisms that prevent the production and diffusion of harmful mediators [18]. These observations are consistent with the levels of AEA and 2-AG observed in this study. In particular, we found higher AEA and 2-AG levels in SHRs despite an increase in FAAH activity, which is the primary enzyme responsible for AEA degradation [9]. Changes in endocannabinoid levels, which are cannabinoid receptor ligands, are accompanied by a decrease in the expression of the CB_2_ receptor. The CB_2_ receptor is responsible for reducing levels of ROS and TNFα [9], which confirms the shift of metabolic processes towards oxidative processes and intensification of inflammatory processes in the brain tissue of SHRs. Therefore, the FAAH inhibitor URB597 may be a useful potential therapeutic compound for counteracting the effects of hypertension.

Administration of URB597 was associated with a significant reduction in FAAH activity within brain tissue. This reduction in FAAH activity resulted in a substantial increase in AEA levels in hypertensive animals, and a smaller increase in AEA in control animals. Arachidonic acid is the primary precursor of a wide range of lipid mediators generated during enzyme-dependent metabolism, including the two major endocannabinoids in the brain (i.e., AEA and 2-AG). Significantly increased endocannabinoid levels in SHR brain tissue following URB597 administration may be associated, in part, with the activation of phospholipases and the release of arachidonic acid, in particular, and a decrease in phospholipid fatty acids. The simultaneous reduction in levels of MDA, which is a product of oxidative modification of arachidonic acid, may indicate enhanced enzymatic metabolism with the generation of endocannabinoids.

Interestingly, we observed an increased generation of endocannabinoids and an increase in the levels of free fatty acids in the URB597 group as compared with the group of hypertensive rats. Thus, administration of the FAAH inhibitor not only reduces the level of AEA degradation, but also affects the expression of other enzymes involved in the metabolism of phospholipids, including phospholipases [55]. These additional effects may explain the involvement of URB597 in the broadly understood phospholipid metabolism, not only endocannabinoid metabolism. The consequences of such FAAH inhibitor activity could change the view of its pharmacological significance.

Unfortunately, we found that URB597 administration was also accompanied by a further decrease in CB_2_ receptor expression, which may reflect a further intensification of oxidative conditions. However, chronic use of the FAAH inhibitor results in a pronounced upregulation of AEA, which activates the GPR55 receptor. Thus, unexpected changes in redox balance are anticipated following FAAH inhibitor administration. GPR55 upregulation is known to inhibit NADPH oxidase activation, thereby limiting ROS production [56]. In addition, GPR55 can also generate heterodimers with other receptors, including CB_2_ [57]. Therefore, the observed CB_2_ downregulation results from the interaction between GPR55 and CB_2_, rather than a weakening of the action of AEA. Moreover, treatment with H_2_O_2_ was found to stimulate HT22 neuronal cells with AEA. This treatment resulted in the suppression intracellular levels of ROS and the Nox2 protein, whereas the reverse was observed following the use of the CB_1_ antagonist AM251 or CB1-siRNA [22]. In addition, prior research demonstrated that AEA can protect neurons from oxidative stress and hypoxia by activating CB_1_ [58]; however, we found no significant changes in CB_1_ receptor expression in the present study. In addition, other studies have demonstrated that under oxidative conditions, AEA promotes increased SOD and GSH activity through CB_1_ activation that results from the inhibition of NOX2 [22].

The results of the present study demonstrate that chronic inhibition of FAAH activity is conducive to increased activity of antioxidant enzymes. This increased enzymatic activity may result from the reduced generation of ROS. Reduced ROS may be due, in turn, to overexpression of the GPR55 receptor, and, consequently, reduced possibilities for protein oxidation. Another mechanism affecting the upregulation of antioxidant enzyme activity is the possible increase in the transcription efficiency of Nrf2. The chronic use of URB597 reduces the expression of the Nrf2 Bach1 nuclear inhibitor, which results in increased transcription activity of Nrf2 and the consequences of upregulation of cytoprotective proteins including antioxidant enzymes. Similar results were observed in studies on breast cancer cells [59].

## 5. Conclusions

This study demonstrates that AEA modifies the redox balance of brain cells by reducing oxidative phospholipid modifications. AEA works by directly acting on the antioxidant system and indirectly by activating G protein-associated receptors. Therefore, an antioxidant response is initiated in brain tissue following the introduction of the FAAH inhibitor URB597. In previous studies, we found that the FAAH inhibitor URB597 exerted a pro-oxidative effect on the heart, liver, kidneys, and blood plasma of rats with primary and secondary hypertension. However, the results of this work show the opposite effect of URB597, which reveals the antioxidant effect on brain tissue. This is the first information about the FAAH inhibitor’s tendency to shift the redox balance towards a reduction reaction in the brain of hypertensive rats. Consequently, this study points to the endocannabinoid system as a potential therapeutic target for preventing metabolic disorders in the brain in hypertension.
